# A Screen for Endocytic Motifs

**DOI:** 10.1111/j.1600-0854.2010.01056.x

**Published:** 2010-04-05

**Authors:** Patrycja Kozik, Richard W Francis, Matthew N J Seaman, Margaret S Robinson

**Affiliations:** 1 University of Cambridge, Cambridge Institute for Medical Research, Wellcome Trust/MRC BuildingHills Road, Cambridge CB2 0XY, UK; 2 Telethon Institute for Child Health Research, Centre for Child Health Research, The University of Western AustraliaPO Box 855, West Perth, WA 6872, Australia

**Keywords:** AP-2, cargo, clathrin, endocytosis, sorting signals

## Abstract

Sorting signals for cargo selection into coated vesicles are usually in the form of short linear motifs. Three motifs for clathrin-mediated endocytosis have been identified: YXXΦ, [D/E]XXXL[L/I] and FXNPXY. To search for new endocytic motifs, we made a library of CD8 chimeras with random sequences in their cytoplasmic tails, and used a novel fluorescence-activated cell sorting (FACS)-based assay to select for endocytosed constructs. Out of the five tails that were most efficiently internalized, only one was found to contain a conventional motif. Two contain dileucine-like sequences that appear to be variations on the [D/E]XXXL[L/I] motif. Another contains a novel internalization signal, YXXXΦN, which is able to function in cells expressing a mutant µ2 that cannot bind YXXΦ, indicating that it is not a variation on the YXXΦ motif. Similar sequences are present in endogenous proteins, including a functional YXXXΦN (in addition to a classical YXXΦ) in cytotoxic T-lymphocyte-associated protein 4 (CTLA-4). Thus, the repertoire of endocytic motifs is more extensive than the three well-characterized sorting signals.

Endocytosis is essential for processes such as nutrient uptake, synaptic transmission, and the immune response. The major endocytic pathway in mammalian cells is mediated by clathrin-coated vesicles (CCVs) (1,2). Selection of cargo into CCVs relies on internalization signals in the cytoplasmic tails of membrane proteins. Internalization signals are usually in the form of short linear motifs, and they work by binding transiently to proteins and protein complexes called adaptors. The adaptors also interact with clathrin and with each other, and thus provide a link between the cargo and the clathrin coat.

There are three motifs that have been shown to be both necessary and sufficient for the uptake of cargo into CCVs: YXXΦ (where Φ is a bulky hydrophobic residue), [D/E]XXXL[L/I] and FXNPXY. The YXXΦ and [D/E]XXXL[L/I] motifs are both recognized by the most abundant adaptor in plasma membrane-derived CCVs, the adaptor protein-2 (AP-2) complex, which is a heterotetramer composed of α, β2, µ2 and σ2 subunits. YXXΦ motifs bind to the µ2 subunit, while [D/E]XXXL[L/I] motifs bind mainly to the σ2 subunit, with an additional contribution from the α subunit. FXNPXY motifs interact with the phosphotyrosine-binding (PTB) domains of some of the ‘alternative adaptors', including Dab1, Dab2, ARH and Numb (3). The structural basis for all three types of interactions has now been established by X-ray crystallography (4–6).

It remains unanswered whether the YXXΦ, [D/E]XXXL[L/I] and FXNPXY signals constitute the full repertoire of endocytic motifs. There are several examples of proteins that are efficiently internalized without any of these signals, such as several of the post-Golgi SNAREs (7). However, it is possible that packaging of such proteins into CCVs could be mediated by a folded domain (as in the case of the SNAREs vti1b and VAMP7 and their adaptors epsinR and Hrb) (8,9), by ubiquitination (3,10), or, if the protein is part of a complex, by interactions with one or more of the other subunits. Here, we have applied an unbiased screening approach using cultured cells to search for novel endocytic motifs in CD8 chimeras. Among the motifs we identified are two variations on the [D/E]XXXL[L/I] motif and one novel tyrosine-based motif.

## Results

### Validation of the system

**Figure 1:**
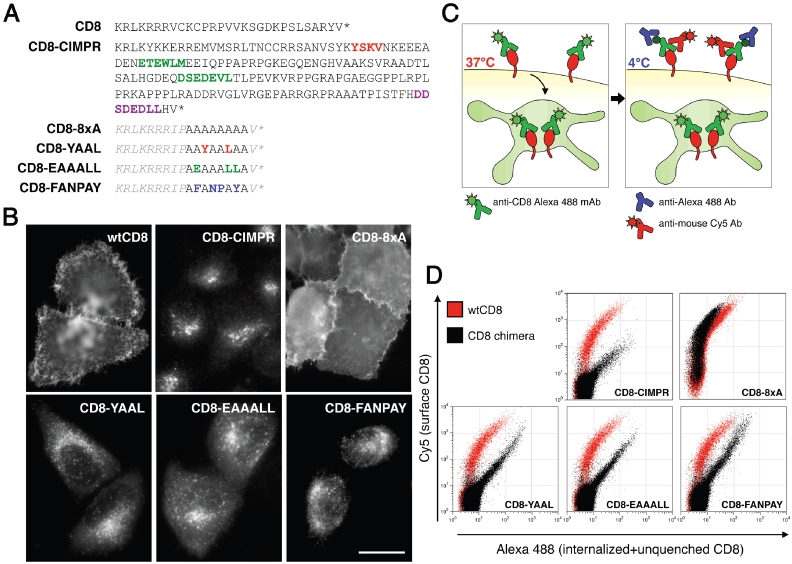
A CD8 reporter-based system to study internalization signals A) Cytoplasmic tail sequences of wtCD8, CD8 with the cytoplasmic tail of CIMPR, and the four ‘designer’ CD8 chimeras. Known trafficking motifs are indicated in colour. B) HeLa cells were transfected with the six constructs listed in (A), and the steady-state localization of the constructs was examined by immunofluorescence microscopy. Scale bar: 20 µm. C) Schematic representation of the assay used to measure the efficiency of internalization of CD8 chimeras. Cells are harvested 24 h post-transfection and allowed to internalize anti-CD8 conjugated to Alexa Fluor 488 for 40 min at 37°C. The cells are then shifted to 4°C and surface Alexa Fluor 488 is quenched with an antibody against the fluorescent dye. Surface anti-CD8 is also relabelled with a Cy5-conjugated anti-mouse IgG. The cells are analysed by flow cytometry. D) FACS profiles of cells transiently transfected with the six CD8 constructs.

The CD8 chimera system has been used to study sorting signals in the cytoplasmic tails of type I transmembrane proteins for over 20 years (11), including proteins that are trafficked in a clathrin-dependent manner such as the cation-independent mannose 6-phosphate receptor (CIMPR) (12–15). Our aim was to adapt the CD8 chimera system to screen short sequences of random amino acids for new internalization signals. We started by generating four chimeras with very simple ‘designer’ tails to act as reference points, because often naturally occurring membrane proteins contain more than one sorting signal (e.g. the CIMPR has at least four) (Figure 1A). All four designer tails contain eight amino acids between common proline and valine residues: either eight alanines (8xA) or with two to four of the alanines replaced by other residues. Thus, the YXXΦ, [D/E]XXXL[L/I] and FXNPXY motifs are represented by YAAL, EAAALL and FANPAY, respectively (Figure 1A). The four designer constructs, as well as wild-type CD8 and a CD8-CIMPR chimera, were transfected into HeLa cells and their steady-state localization was examined by immunofluorescence. CD8-YAAL, CD8-EAAALL and CD8-FANPAY all have a punctate intracellular pattern (Figure 1B) and partially colocalize with the endosome and lysosome markers EEA1 and CD63 (Figure S1). In contrast, CD8-8xA, like wild-type CD8, is found mainly at the cell surface.

To measure how efficiently the constructs are internalized, we developed a novel FACS-based antibody uptake assay (Figure 1C). Transiently transfected cells were first allowed to internalize anti-CD8 labelled with Alexa Fluor 488 for 40 min, and then shifted to 4°C to stop further trafficking. To distinguish between internalized and surface-bound antibody, surface fluorescence was quenched using an anti-Alexa Fluor 488 antibody. In addition, because quenching is never 100% (Figure S2), surface anti-CD8 was also labelled with a Cy5-conjugated secondary antibody. The FACS profiles of cells transfected with wtCD8 and CD8-8xA are very similar (Figure 1D), with a high red-to-green ratio, whereas the FACS profiles of cells transfected with CD8-CIMPR, CD8-YAAL, CD8-EAAALL and CD8-FANPAY all show a higher green-to-red ratio than wtCD8, at all expression levels.

### Generation and screening of a CD8 library

**Figure 2:**
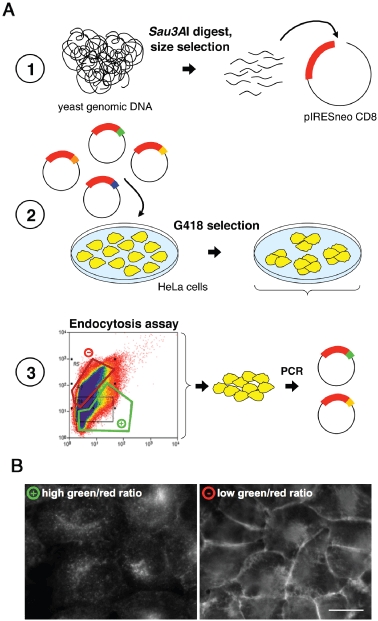
A CD8 library-based approach to identify novel endocytic motifs A) Schematic representation of the approach. (1) Size-selected fragments of yeast genomic DNA are ligated into a CD8-encoding plasmid and transformed into bacteria to generate the library. (2) HeLa cells are transfected with the library and stably transfected cells are selected with G418. (3) The stable transfectants are pooled and endocytosis efficiency is assayed by FACS (see Figure 1). Cells with a high green-to-red fluorescence ratio (green gate, indicated by a plus sign) and with a low green-to-red fluorescence ratio (red gate, indicated by a minus sign) are collected, and tail sequences are determined using a cell-based PCR method. B) Steady-state distribution of the constructs in the two populations of cells. Scale bar: 20 µm.

To identify new short motifs that might act as internalization signals, we generated a library of CD8 chimeras with short random sequences in their cytoplasmic tails. The random sequences were derived from a size-selected *Sau3A*I digest of yeast genomic DNA (Figure 2A). About 20 000 bacterial colonies were pooled together and their plasmids were isolated and transfected into HeLa cells. After G418 selection for stable transfectants, the cells were harvested and the antibody uptake assay was carried out using preparative flow cytometry.

Two populations of cells were collected: cells with a high ratio of green-to-red fluorescence, indicated with a ‘+’ sign in Figure 2A and predicted to be expressing constructs with internalization signals; and cells with a low ratio of green-to-red fluorescence, indicated with a ‘−’ sign and predicted to be expressing constructs without internalization signals. Immunofluorescence labelling confirmed that the steady-state localization patterns of the CD8 chimeras in the two populations of cells were intracellular and plasma membrane, respectively (Figure 2B).

Next, we used polymerase chain reaction (PCR) to amplify the tails of the CD8 chimeras from the cells in the ‘+’ population, recloned them into the CD8 vector, and determined their sequences. The endocytosis efficiency of the individual constructs was assayed again by flow cytometry (Figure S3).

**Figure 3:**
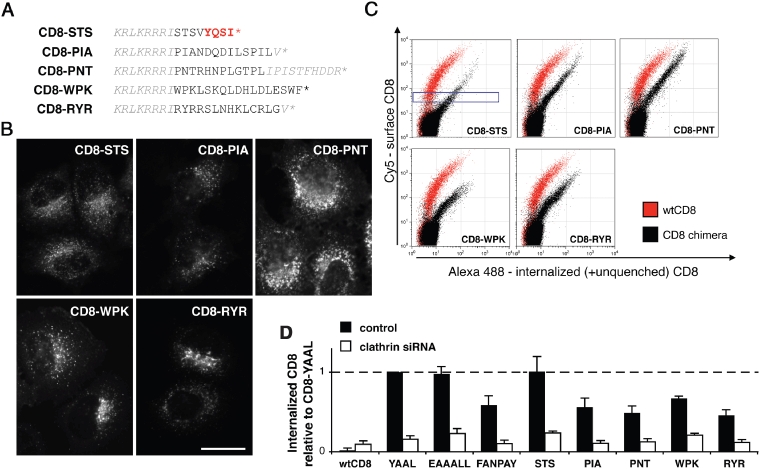
CD8 chimeras with internalization signals isolated from the library A) Sequences of the five cytoplasmic tail inserts that promoted the most efficient internalization of anti-CD8. Vector sequences are shown in italics. The nomenclature refers to the first three residues of the insert. B) Steady-state localization of transiently transfected constructs. Scale bar: 20 µm. C) To determine the efficiency of internalization of the library constructs, the antibody uptake assay was carried out on transiently transfected cells. D) Bar graphs showing the amount of internalized antibody (mean fluorescence of Alexa Fluor 488) for cells expressing equal amounts of surface CD8 [gated as shown in (C) for CD8-STS].

The sequences of the five tails that were internalized most efficiently are shown in Figure 3A. Surprisingly, only one of the sequences contains a classical internalization motif, a YXXΦ (YQSI) in CD8-STS (the nomenclature refers to the first three residues in the tail insert). As expected, all five constructs have a mainly intracellular steady-state distribution (Figure 3B). To compare the endocytosis efficiency of the five library constructs with that of the designer constructs, we quantified the amount of internalized anti-CD8 antibody (mean Alexa Fluor 488 fluorescence) at a relatively low level of surface expression (defined by the blue gate in Figure 3C; the same gate was used for all of the constructs). CD8-YAAL, CD8-EAAALL and the library clone CD8-STS were all internalized with similar efficiencies to each other, while the internalization efficiency of CD8-FANPAY and of library clones CD8-PIA, CD8-PNT, CD8-WPK and CD8-RYR was about half that of CD8-YAAL, but still much greater than that of wtCD8 (Figure 3D).

To determine whether internalization of the constructs is clathrin dependent, we used siRNA to deplete clathrin heavy chain from the cells (16) (Figure S4). In the clathrin-depleted cells, endocytosis of all the library constructs was essentially abolished (Figure 3D). Thus, we have identified four short sequences that do not conform to known internalization signals, but that promote clathrin-mediated endocytosis of the CD8 reporter.

### Variations on the [D/E]XXXL[L/I] motif

Do the random tails mimic known motifs, or do they contain new motifs? To determine which residues are important for endocytosis, we mutated individual amino acids and then assayed the phenotype.

**Figure 4:**
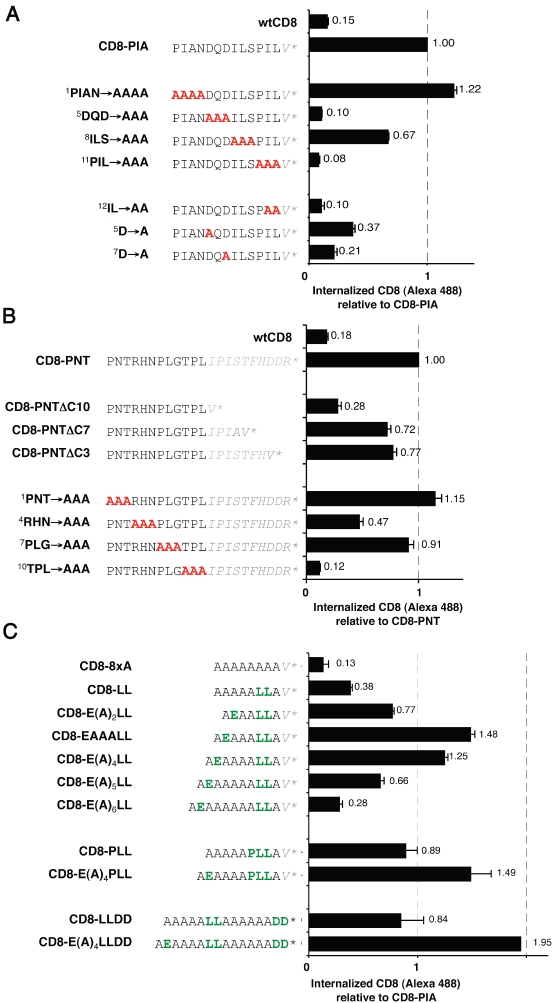
Mutational analysis of CD8-PIA and CD8-PNT suggests that they contain atypical dileucine motifs A) To identify the residues in CD8-PIA that mediate its internalization, the amino acids in the insert were mutated in groups of three or four. The mutated constructs were expressed in HeLa cells and the antibody uptake assay was carried out. B) Mutational analysis of CD8-PNT. To determine which residues from the vector sequence (in italics) contribute to the internalization of CD8-PIA, C-terminally truncated constructs were studied. Alanine-scanning mutagenesis was then carried out on the full-length tail. C) Internalization of designer constructs with variations on acidic dileucine motifs.

We first investigated the tail of CD8-PIA by replacing three or four residues at a time with alanines (Figure 4A). Both the ^5^DQD and the ^11^PIL mutation completely abolished internalization, while the ^8^ILS mutation had a partial effect. These data suggested that endocytosis of CD8-PIA might be mediated by an unconventional acidic dileucine motif(s). The classical dileucine motif has three amino acids between the acidic residue and the dileucine, whereas CD8-PIA contains two IL sequences, with acidic residues at positions −1, −3, −5 or −7. Additional mutagenesis indicated that the second IL sequence is most important, and that the second aspartic acid (D7, at position −5 relative to the second IL) is more important than the first.

Unlike the other constructs, CD8-PNT required the vector sequence as well as the library sequence for efficient internalization (Figure 4B). As the vector sequence on its own does not confer internalization (Figure S5), we reasoned that the internalization signal might incorporate residues encoded by both the vector and the insert. Deletion mutagenesis indicated that both the IPI sequence and the DDR sequence of the vector contribute to the internalization signal. When we performed alanine-scanning mutagenesis on the full-length tail, we found that mutation of ^4^RHN inhibited and ^10^TPL abolished the efficient internalization of the construct. Hence, the critical residues for CD8-PNT internalization appear to be contained within the TPL and IPI triplets, suggesting that like CD8-PIA, CD8-PNT might contain a variation on the dileucine motif.

To test how such variations might affect the efficiency of endocytosis, we made additional designer constructs, modifying the original CD8-EAAALL construct by adding or removing alanine residues between the E and the LL. The internalization efficiencies of the constructs are shown in Figure 4C. Interestingly, all of the constructs were internalized more efficiently than wtCD8 or CD8-8xA. Although the original construct was internalized with the greatest efficiency, a chimera with four spacer residues was internalized nearly as efficiently, and chimeras with two or five spacer residues were internalized about half as efficiently. Even a dileucine on its own was able to act as a weak internalization signal.

Both CD8-PIA and CD8-PNT have a proline residue just upstream from the dileucine. This type of sequence is found in several endogenous proteins with dileucine motifs (3) and there is some evidence that a proline in this position improves the binding of the motif to AP complexes (17,18). Similarly, when we made designer constructs with a PLL sequence, we found that the efficiency of endocytosis was at least twofold higher than that of the same construct with an ALL sequence (Figure 4C; cf. CD8-PLL with CD8-LL and CD8-E(A)_4_PLL with CD8-E(A)_5_LL). We also investigated the role of the downstream aspartic acid pair found in CD8-PNT, because again, such sequences are found in naturally occurring proteins and have been implicated in AP-2 binding and endocytosis (19). A construct with a DD sequence six residues downstream from the dileucine (CD8-LLDD) was internalized about twice as efficiently as the same construct without the DD (CD8-LL). Moreover, when we replaced the alanine in this construct at position −5 with glutamic acid to make CD8-E(A)_4_LLDD, the construct was internalized more efficiently than the corresponding constructs with only upstream (CD8-E(A)_4_LL) or downstream (CD8-LLDD) acidic residues. Thus, upstream and downstream acidic residues can act synergistically to improve the internalization efficiency of dileucine-containing proteins.

### CD8-WPK

**Figure 5:**
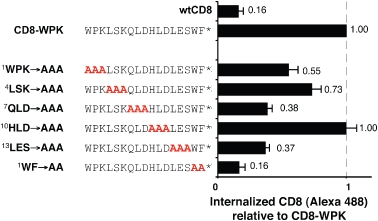
Mutational analysis of CD8-WPK Alanine-scanning mutagenesis was carried out to identify the residues involved in the internalization of CD8-WPK.

Unlike the other constructs, CD8-WPK contains neither tyrosines nor dileucine-like motifs. Alanine-scanning mutagenesis of CD8-WPK showed that internalization was inhibited most strongly when the C-terminal WF was mutated. In addition, ^4^LES, ^7^QLD, and to a lesser extent ^1^WPK and ^4^LSK residues appeared to be important for the internalization (Figure 5). Together, these data suggest that CD8-WPK might interact with the clathrin machinery using more than one anchor point rather than a simple internalization signal, and the construct was not pursued further.

### A novel tyrosine-based internalization signal

**Figure 6:**
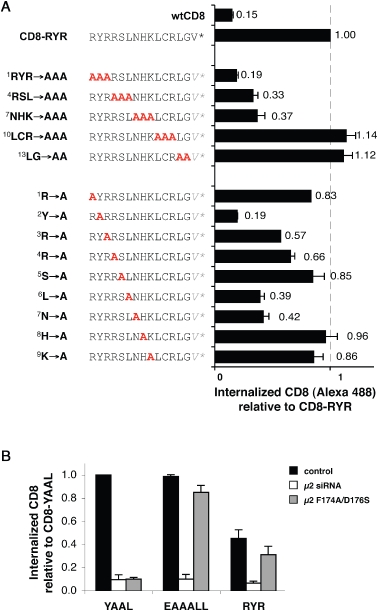
CD8-RYR contains a novel tyrosine-based motif A) Alanine-scanning mutagenesis was carried out to identify the residues involved in the internalization of CD8-RYR. The most important residues are Y2, L6 and N7. B) Endocytosis of CD8-RYR in cells expressing µ2 with a mutation (F174A/D176S) in the tyrosine-binding pocket. Wild-type HeLa cells, or HeLa cells expressing siRNA-resistant µ2 F174A/D176S, were depleted of endogenous µ2, transfected with the CD8 constructs and processed for the antibody uptake assay. The mutant µ2 rescues the uptake of CD8-EAAALL and CD8-RYR, but not of CD8-YAAL.

Alanine-scanning mutagenesis of the CD8-RYR tail showed that in three of the mutants, ^1^RYR, ^4^RSL and ^7^NHK, efficient endocytosis was either abolished or significantly impaired (Figure 6A). To identify amino acids within this region that are important for internalization, we mutated individual residues between R1 and K9 to alanines. We found the strongest decrease in endocytosis efficiency in constructs with mutations in Y2, L6 and N7, with the tyrosine being critical for internalization.

Initially, we suspected that like CD8-PIA and CD8-PNT, CD8-RYR might contain a variation on a known motif, because YXXXLN is similar to YXXΦ but with an extra amino acid between the tyrosine and the bulky hydrophobic residue. Moreover, it has been shown that the internalization signal of the P2X2 receptor, YXXGΦ, binds to µ2 using the same sites as a YXXΦ motif (20). However, structural predictions indicate that unlike glycine, a serine in the +3 position would inhibit the binding of the hydrophobic residue to µ2 (David Owen, personal communication). To test experimentally whether CD8-RYR is recognized by the YXXΦ binding site of µ2, we used a cell line that stably expresses an siRNA-resistant version of µ2 with a mutation (F174A/D176S) in the tyrosine binding pocket. This mutation both abolishes binding to the YXXΦ motif *in vitro* and fails to rescue transferrin internalization *in vivo*(21,22). Depleting endogenous µ2 (Figure S4) in control cells prevents internalization of the two AP-2-dependent designer proteins, CD8-YAAL and CD8-EAAALL, as well as of CD8-RYR (Figure 6B). When endogenous µ2 is depleted in cells that stably express the F174A/D176S µ2 mutant, the mutant construct completely fails to rescue the uptake of CD8-YAAL, but does rescue the uptake of both CD8-EAAALL and CD8-RYR. Because endocytosis of CD8-RYR is critically dependent on the tyrosine residue, these data suggest that the YXXXLN sequence is not a variation on the YXXΦ motif, but is instead a novel tyrosine-based internalization signal.

### Sorting signals in endogenous proteins

If YXXXLN is an internalization signal, then there might be endogenous proteins that contain the same motif. To search for this motif in the cytoplasmic tails of human proteins, we generated a database of the tail sequences of predicted single pass transmembrane proteins (available at http://www.bioinformatics.cimr.cam.ac.uk/transtype/). About 50% of type I transmembrane proteins (649 out of 1266) do not contain sequences conforming to any of the three well-characterized internalization signals, supporting the notion that alternative motifs might exist. Interestingly, a relatively high proportion of the proteins contain more than one internalization signal. Seventy-three of the proteins with a [D/E]XXXL[L/I], and 78% of the proteins with an FXNPXY, also have a YXXΦ, compared with 47% of the proteins overall; and 67% of the proteins with an FXNPXY also have a [D/E]XXXL[L/I], compared with 15% of the proteins overall. Most strikingly, all nine of the proteins with a Golgi-localized, γ-ear-containing, ADP ribosylation factor (ARF)-binding protein (GGA)-dependent sorting signal, defined as a DXXLL motif within three residues of the C-terminus, also have a YXXΦ and/or [D/E]XXXL[L/I] motif. For comparison, not one of the seven proteins with the endoplasmic reticulum (ER)-retrieval signal KKXX has a classical internalization signal.

**Figure 7:**
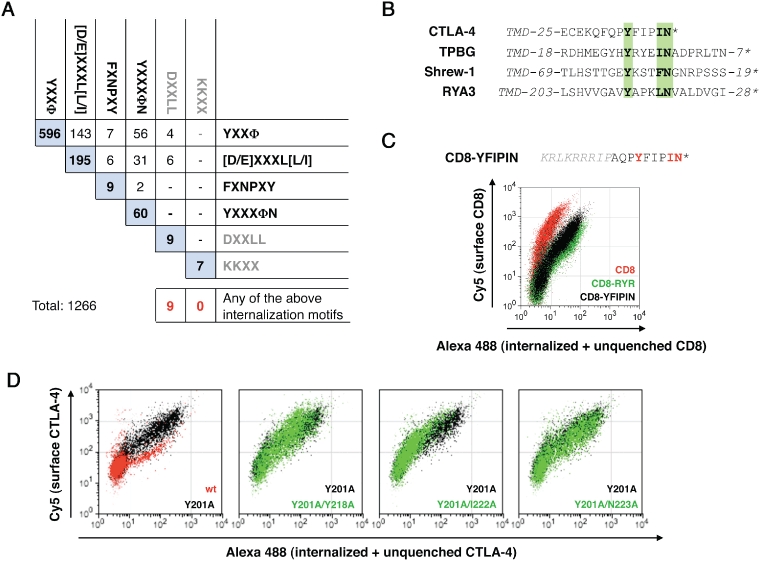
Sorting signals in endogenous proteins A) To search for trafficking motifs in naturally occurring proteins, we generated a database of 4794 cytoplasmic tail sequences of single-pass transmembrane proteins (1266 type I and 3528 type II proteins). We then searched the database for tails from type I proteins that contain known endocytic motifs (YXXΦ, [D/E]XXXL[L/I], FXNPXY) or our candidate novel endocytic motif (YXXXΦN), as well as motifs recognized by GGAs and COPI (DXXLL and KKXX, respectively). The table lists the frequency of the various sorting signals. The same sorting signals are shown on both the *x*- and *y*-axis. Where a sorting signal on the *x*-axis intersects with the same sorting signal on the *y*-axis, the number in the (blue) box is the number of proteins with at least one copy of that motif. Where a sorting signal on the *x*-axis intersects with a different sorting signal on the *y*-axis, the number in the box is the number of proteins that have both motifs (e.g. there are 143 proteins with both YXXΦ and [D/E]XXXL[L/I] motifs). The red numbers indicate how many of the nine tails with the post-Golgi motif, DXXLL, or of the seven tails with the ER-retention motif, KKXX, also contain internalization signals (YXXΦ, [D/E]XXXL[L/I], FXNPXY or YXXXΦN). The database can be searched for known or novel motifs and is accessible online at http://www.bioinformatics.cimr.cam.ac.uk/transtype/. B) Examples of type I membrane proteins with potential YXXXΦN motifs. The numbers of residues in the cytoplasmic tails both before and after each sequence are indicated. C) A C-terminal fragment of the cytoplasmic tail of CTLA-4 bearing a YXXXΦN motif was transplanted onto CD8, and the antibody uptake assay was carried out. D) Full-length CTLA-4, either wild-type or with the indicated mutations, was transiently transfected into HeLa cells and the antibody uptake assay was carried out using anti-CTLA-4 instead of anti-CD8.

When we looked for the sequence YXXXΦN in our database of type I membrane protein tails, we found 60 examples (Table S1), some of which are shown in Figure 7B. Again, many of the proteins with a YXXXΦN sequence also contain additional internalization signals: 93% have a YXXΦ, 52% have a [D/E]XXXL[L/I] and 3% have an FXNPXY. To test whether the YXXXΦN sequences from endogenous proteins can mediate internalization, we transplanted the eight C-terminal residues from cytotoxic T-lymphocyte antigen-4 (CTLA-4) onto the CD8 reporter. CTLA-4 has a classical YXXΦ motif, and it has been shown that mutating the tyrosine in this motif increases the surface expression of CTLA-4. However, when the tyrosine near the C-terminus is also mutated, there is a further increase in surface expression, suggesting that this tyrosine is part of a second internalization signal (23,24). We found that when we made a CD8 chimera in which we replaced the last eight residues of a designer tail with the last eight residues of CTLA-4, QPYFIPIN, the construct was internalized with a similar efficiency to CD8-RYR.

We also did the converse experiment, mutating the YFIPIN sequence in full-length CTLA-4. Because CTLA-4 is a protein normally expressed only in T-lymphocytes, with well-characterized antibodies against its extracellular domain, we were able to use the same flow cytometry-based antibody uptake assay that we used on the CD8 chimeras. Mutating the first tyrosine in the CTLA-4 tail, which is part of the YXXΦ motif, caused an increase in the ratio of red-to-green fluorescence, indicating that internalization is less efficient. Mutating the second tyrosine, in the YFIPIN sequence, caused a slight further increase in the ratio of red-to-green fluorescence, consistent with previous work (24) (Figure 7D). A more substantial increase was observed when we mutated the isoleucine residue, indicating that it also contributes to the internalization signal. In contrast, the red-to-green fluorescence ratio was unchanged when we mutated the asparagine residue, indicating that at least in the context of CTLA-4, this residue is less important. Nevertheless, the contribution of both the tyrosine and the isoleucine to CTLA-4's second internalization signal shows that our approach of screening a random tail library for efficiently internalized constructs, followed by database searching for naturally occurring transmembrane proteins that have similar sequences in their cytoplasmic tails, can be used to identify new endocytic motifs.

### Role of AP-2 in internalization of the library constructs

A key question is whether the library constructs, in particular CD8-WPK and CD8-RYR, use AP-2 as an endocytic adaptor or whether they use alternative adaptors. Interactions between adaptors and sorting signals are relatively weak, with *K*_*d*_s in the micromolar range (1), so it can be difficult to demonstrate binding directly. Two methods that have proved useful in the past are yeast two- or three-hybrid assays (25) and surface plasmon resonance (21); however, neither method revealed any binding of our library constructs to AP-2.

**Figure 8:**
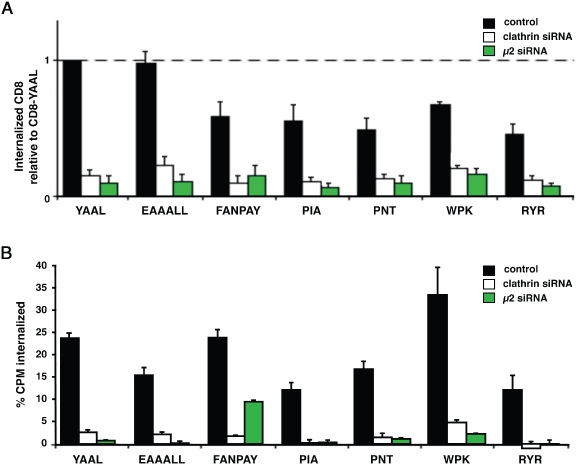
Effect of AP-2 depletion on endocytosis A) Antibody uptake assay carried out to compare the effects of clathrin depletion and AP-2 depletion on the various constructs. B) Uptake of the same constructs investigated using a different assay. Cells were stably transfected with the constructs and antibody followed by ^125^I-labelled protein A was prebound to the cells at 4°C, then the cells were warmed to 37°C for 10 min to measure a single round of endocytosis.

A less direct but still informative method is to deplete AP-2 with siRNA and then to observe the phenotype. Using our flow cytometry-based assay, we found that endocytosis of CD8-PIA, CD8-PNT, CD8-WPK and CD8-RYR was strongly inhibited by AP-2 knockdown, similar to what we found when we carried out a clathrin knockdown (Figure 8A). However, endocytosis of all three of our designer constructs was also strongly inhibited, including CD8-FANPAY, which does not bind directly to AP-2. It has been shown that the effect of AP-2 depletion on endocytosis mediated by alternative adaptors varies widely according to the assay used. When a steady-state type of assay is used to measure endocytosis, all CCV cargo proteins are affected, whether or not they interact directly with AP-2, presumably because there are many fewer clathrin-coated pits at the plasma membrane in AP-2-depleted cells (26). However, when a ligand is prebound to the plasma membrane at 4°C and a single round of endocytosis is measured, AP-2 depletion affects cargo proteins that bind directly much more strongly than those that use alternative adaptors (16,27). This is presumably because although the number of coated pits goes down ∼10-fold, the relevant adaptors are still present, and the cargo proteins (which no longer have to compete with other cargo proteins that depend upon AP-2) are efficiently recruited into these pits at 4°C and are then rapidly endocytosed when the cells are warmed up. Because our flow cytometry-based assay measures multiple rounds of endocytosis, we investigated the same set of constructs using a prebinding assay.

For these experiments, we first had to generate stably transfected cell lines for both the library constructs and the designer constructs. Cells expressing each of these constructs were allowed to bind anti-CD8 followed by ^125^I-labelled protein A at 4°C. They were then washed and warmed to 37°C for 10 min. Label remaining on the cell surface was removed with an acid wash, and both surface and internalized label were quantified. Figure 8B shows the percentage of the total prebound label that was internalized. As expected, endocytosis of both of the two designer constructs that bind to AP-2, YAAL and EAAALL, was strongly inhibited by knocking down AP-2. However, the effect of AP-2 depletion on CD8-FANPAY was much weaker. Interestingly, all four of the library constructs were strongly affected by knocking down AP-2, indicating that they all depend upon AP-2 for their internalization.

## Discussion

The aim of this project was to test in an unbiased way whether the repertoire of known internalization signals is complete, or whether there are other motifs yet to be characterized. In a library screen for endocytosed constructs, we found four sequences that did not conform to any of the known motifs, but were capable of promoting efficient internalization of CD8. Through a mutational analysis of these constructs, we were able to identify the key residues. We found that variations on the [D/E]XXXL[L/I] motif can act as internalization signals in the context of the CD8 reporter and are abundant in endogenous proteins. We also found additional motifs that appear to be distinct from the known repertoire. Further characterization of one of the tails narrowed down the internalization signal to YXXXΦN, a motif also found in endogenous proteins such as CTLA-4.

Although at the time that we carried out these experiments, there was no structural information on how acidic dileucines interact with AP complexes; the crystal structure of the AP-2 core with a bound dileucine peptide has since been published (6). Our observations are entirely in agreement with the crystallography data. For instance, the crystal structure shows that the interaction between AP-2 and the dileucine motif is mediated mainly by two hydrophobic pockets in the σ subunit of AP-2, which accommodate the two leucine residues of the peptide. A lesser contribution is made by the Y-4 residue of the peptide, which interacts electrostatically with a positively charged patch on AP-2. Similarly, here we show that a dileucine on its own can act as a weak internalization signal, but that an upstream acidic residue increases the efficiency of endocytosis. However, this residue does not need to be at the −4 position; an acidic residue at the −5 position works nearly as well. This is consistent with the crystal structure, which shows that the patch on AP-2 could accommodate more distant residues as well as those in the −4 position. These residues do not necessarily have to be acidic: the peptide used for crystallization had a glutamine at the −4 position, and the affinity of binding was shown to be only slightly higher when the glutamine was replaced by a glutamic acid. Likewise, the upstream residues that contribute to the endocytosis of CD8-PNT, RHN, are hydrophilic but not acidic. Our findings are also consistent with the observations that have been made on naturally occurring proteins with dileucine motifs. Many of these proteins do not have an acidic residue at the −4 position and therefore must be using either another hydrophilic residue (e.g. a phosphoserine or a glutamine) at the −4 position and/or an acidic/hydrophilic residue further upstream (3).

Both CD8-PIA and CD8-PNT have a proline in the −1 position. A proline in this position has been shown to improve the binding of dileucine-containing constructs to both AP-2 and AP-3 *in vitro*(17,18), and there is also an *in vivo* study dating back to 1994, in which mutating the EQLPML internalization signal of major histocompatibility complex II (MHC II) invariant chain to EQLAML greatly reduced its rate of endocytosis (28). The crystal structure of AP-2 with a bound dileucine peptide again provides an explanation for these findings, because it shows that a proline in the −1 position would increase the strength of binding by altering the conformation of the peptide backbone (6).

Downstream acidic residues can also improve the efficiency of endocytosis. While this manuscript was in preparation, Bonifacino and coworkers showed that a pair of acidic residues (DD) downstream from a dileucine are critical for the interaction of Nef, an HIV-1-encoded protein, with AP-2 (19), and they subsequently showed that a second basic patch on AP-2, which was revealed by the crystal structure, interacts with these downstream residues. (29). Similarly, CD8-PNT has a downstream DD sequence, and we found that inserting a DD into designer constructs downstream from the dileucine caused them to be internalized more efficiently. Thus, although we carried out our analyses without knowing the structural basis for dileucine recognition, our observations fit together with the crystallography data remarkably well, supporting the validity of both the random tail approach and our endocytosis assay.

Unlike the two constructs with dileucine-like motifs, the tyrosine-containing construct CD8-RYR appears to use a different molecular mechanism from the two known tyrosine-based motifs, YXXΦ and FXNPXY. The structural basis for the recognition of both of these motifs by their respective adaptors has been established by X-ray crystallography, and it is clear that the CD8-RYR tail would be unable to use the FXNPXY binding site, and unlikely to use the YXXΦ binding site. Further evidence comes from our demonstration that the construct is still efficiently internalized in cells expressing a mutated µ2 that is unable to bind the YXXΦ motif. Thus, we propose that CD8-RYR uses a novel internalization signal, YXXXΦN. YXXXΦN sequences are present in endogenous proteins as well, including CTLA-4, which contains the sequence YFIPIN in its cytoplasmic tail. Using the same flow cytometry-based assay that we established for CD8 chimeras, we were able to investigate the importance of the YFIPIN sequence in CTLA-4, and found that both the tyrosine and the second isoleucine contribute to its internalization.

The library construct that we know least about is CD8-WPK, because the mutagenesis analysis indicates that multiple interactions contribute to its internalization, so it was not pursued any further. However, it is endocytosed extremely efficiently, especially in the prebinding assay shown in Figure 8B, where it was taken up even more rapidly than our designer constructs. Thus, it will be worth further characterizing this construct to try to understand how it is internalized, and to look for similar proteins in nature.

All of our library constructs were strongly affected by AP-2 depletion, especially when we measured the uptake of prebound antibody, indicating that their endocytosis is dependent upon AP-2. However, we cannot necessarily conclude that they all interact directly with AP-2; another possibility is that they interact with an alternative adaptor, which in turn binds to AP-2. For instance, the Drosophila protein Sanpodo requires AP-2 for its internalization, but it has an FXNPXY-like motif and binds to the alternative adaptor Numb, which (unlike most other alternative adaptors) has no clathrin binding site, and thus needs AP-2 in order to engage with the clathrin machinery (30). Further experiments will be needed to find out whether the requirement for AP-2 reflects a direct or an indirect interaction.

Although we size-selected the tails for our library to try to ensure that each one contains only a single internalization signal, our database indicates that many human transmembrane proteins have more than one such signal. This may be important both for fine tuning the rate of internalization and for coordinating endocytosis with intracellular trafficking, because some internalization signals can also act as intracellular sorting signals (e.g. YXXΦ and dileucine motifs can bind to all four AP complexes). Interestingly, we found that all of the proteins in our database with GGA-binding motifs also have either a YXXΦ or a conventional acidic dileucine. It is possible that most—or even all—of the proteins that reside in post-Golgi intracellular compartments need to have internalization signals in case they are missorted to the plasma membrane. In contrast, proteins with the KKXX ER-retrieval motif, which normally never go beyond the early Golgi, do not have internalization signals.

The importance of short linear motifs in protein-protein interactions is only beginning to be appreciated (31,32). These motifs seem to be particularly important for trafficking, and often act as sorting signals to direct cargo into coated vesicles: not only clathrin coated (3,33), but other types as well. For instance, the COPII-coated vesicles that bud from the ER have so far been shown to recognize three distinct motifs, which bind to three independent sites on the Sec24 subunit (34). Several years ago, Roth and coworkers used a similar approach to ours to look for short sequences that promote internalization of influenza virus haemagglutinin, and found several sequences that did not conform to any known motifs (35). These sequences were not analysed further, so it is not clear whether they contained novel motifs, or whether, for instance, some of them might have contained atypical dileucine motifs and/or ubiquitination sites. Nevertheless, given the extensive surface area of the AP-2 complex, together with the existence of multiple alternative adaptors for clathrin-mediated endocytosis, it seems likely that there are additional endocytic motifs that still await discovery.

## Materials and Methods

### Cell lines and tissue culture

The HeLa M cell line (36) was used throughout the study. For some experiments, a cell line was used that stably expresses the F174A/D176S mutant of µ2 (22). The cells were cultured in DMEM (Sigma-Aldrich) supplemented with 10% FBS, l-glutamine and penicillin/streptomycin; for selection and maintenance of stably transfected cell lines, G418 (Gibco BRL) was used at a concentration of 500 mg/mL.

### Plasmids and molecular biology techniques

Standard molecular biology techniques were carried out as described by Sambrook et al. (37). All oligonucleotides were ordered from Sigma-Aldrich, and enzymes were purchased from New England Biolabs. pIRESneo2 vectors expressing CD8 and CD8-CIMPR have been previously described (12). To generate the designer constructs, the CD8-CIMPR vector was digested with *Afl*II and *Not*I, and the inserts were prepared by hybridization and phosphorylation of complementary oligonucleotides.

To generate the random tail library, genomic DNA from a *Saccharomyces cerevisiae* (strain SEY 6210) (38) was digested with *Sau3A*I (which leaves a *BamH*I-compatible overhang), and 50–100 bp fragments were gel purified and ligated into the *BamH*I site (indicated by a dash, Figure S3) of a plasmid encoding CD8 with the cytoplasmic tail sequence KRLKRRRI-PISTFHDDR*. Twenty independent ligation plates, each containing about 1000 colonies, were pooled together to generate the CD8 plasmid library. To determine the complexity of the library, 30 random clones were sequenced. Within these clones, some sequences were overrepresented, most likely because of genomic repeats in *S. cerevisiae*: one was represented twice, two were represented three times and one was represented six times. Thus, out of the 30 clones, there were 20 different sequences, 16 of which were unique, with amino acid frequencies close to those expected based on codon frequencies. Two tail sequences that appeared among the random clones were also later identified within the pool of efficiently internalized chimeras (CD8-RYR, represented six times; CD8-PSR, represented once).

Plasmids were transfected into the cells using Mirus TransIT HeLa MONSTER reagent (Cambridge Bioscience Ltd), according to the manufacturer's instructions (using 1 µg of DNA for a 50% confluent 12-well ). Analysis was performed 24-h post-transfection. For siRNA depletion experiments, the cells were treated with 20 nm siRNAs directed against clathrin heavy chain and µ2 (16) on days 1 and 3, transfected with the CD8 constructs on day 4 and analysed on day 5. The efficiency of clathrin and µ2 depletion was tested by western blotting (Figure S4).

The cytosolic tails of CD8 chimeras expressed in FACS-sorted cells were identified using a cell-based PCR method (37). Briefly, 4 × 10^4^ cells were lysed for 1 h at 37°C in 100 µL of PCR lysis solution [67 mm Tris–Cl at pH 8.8, 16.6 mm ammonium sulfate, 5 mmβ-mercaptoethanol, 6.7 mm MgCl_2_, 6.7 µm ethylenediaminetetraacetic acid (EDTA), 1.7 µm SDS and 50 µg/mL proteinase K], followed by a 10-min incubation at 80°C to inactivate proteinase K. Ten microlitre aliquots of the cell lysates were used in the PCR reaction using HotStarTaq DNA Polymerase (QIAGEN). CD8 chimeras with the insert sequences identified by PCR were generated by hybridization of complementary oligonucleotides as described earlier.

For the experiment shown in Figure 8B, stable cell lines were made by transfecting with the CD8 chimeras and selecting with G418, as previously described (13,16).

### Immunofluorescence and antibody uptake assays

Immunofluorescence was performed on methanol-fixed cells, using anti-CD8 antibody produced by a mouse hybridoma cell line obtained from the American Type Culture Collection (ATCC number: CRL-8014) through LGC Promochem (Teddington). Secondary antibodies (including the antibody against Alexa Fluor 488) were purchased from Molecular Probes. Images were collected using a Zeiss Axiovert 200 inverted microscope fitted with a Zeiss Plan Achromat 63× oil immersion objective, 1.40 numerical aperture (NA), a Hamamatsu Orca ER2 camera and Improvision Openlab software, and they were processed for presentation using Adobe Photoshop.

Before using the library for flow cytometry, random clones were picked and immunofluorescence was carried out. The labelling revealed several different localization patterns, including ER, Golgi, endosomes/lysosomes, a tubular compartment associated with spindle poles during mitosis, and the plasma membrane.

Antibody uptake assays were carried out in round-bottomed 96-well plates, with each well containing 3 × 10^5^ cells. Trypsinized cells were resuspended in growth medium and spun for 2 min at 198 x ***g***. The cells were then incubated at 37°C in growth medium containing Alexa Fluor 488-labelled anti-CD8 (1:50, Serotec). After 40 min, the cells were washed in chilled PBS containing 1% BSA and incubated on ice for 30 min with anti-Alexa Fluor 488 (1:67) Cy5-conjugated anti-mouse immunoglobulin G (IgG) (1:200). The cells were then washed and resuspended either in 3.7% paraformaldehyde (PFA) or (for siRNA-depleted cells) in 1% PBS/BSA containing 7-Aminoactinomycin D (AAD). Analytical flow cytometry was carried out using an FACSCalibur flow cytometer (Becton-Dickinson). For cell sorting, a MoFLow cytometer was used, and the cells were collected into growth medium supplemented with 20% fetal calf serum (FCS). Scatter plots were analysed using FlowJo software (Tree Star). Dead and autofluorescent cells were gated out using the FL-3/FL-1 channels.

For quantification, we chose cells with equal, and relatively low, surface expression of the CD8 construct. This was for two reasons: first, because the efficiency of internalization is likely to decrease with increased expression levels, due to saturation of the machinery (39); and second, because there is less unquenched surface Alexa Fluor 488 in this population of cells. The effect of expression levels on saturation and quenching can be seen in the scatter plots (e.g. Figure 1D). At high expression levels, the profile of cells expressing plasma membrane-associated constructs curves to the right, whereas the profile of cells expressing endocytosed constructs curves to the left. The mean Alexa Fluor 488 fluorescence and SEM were calculated from at least three independent experiments and are presented as bar graphs.

For the experiments shown in Figure 7D, we used anti-CTLA-4 instead of anti-CD8. The antibody (BNI3) was purchased from Abcam and labelled with a Zenon Alexa Fluor 488 Mouse IgG2a Labeling Kit (Invitrogen). It was then diluted 1:50 in growth medium and the endocytosis efficiency assay was carried out as described for anti-CD8.

Uptake of ^125^I-labelled anti-CD8 was carried out as previously described (13,16). Briefly, cells were allowed to bind anti-CD8 followed by ^125^I-protein A at 4°C. The cells were then washed and warmed to 37°C for 10 min. The medium was collected, antibody remaining at the cell surface was removed with a low pH wash, and the cells were extracted with 1 m NaOH. Counts in the medium, in the acid wash and in the cell extract were quantified with a gamma counter.

### Bioinformatics

Data files for both human and mouse were obtained from the LOCATE (PMID: 17986452) website (http://locate.imb.uq.edu.au/downloads.shtml) (40,41). These XML files were parsed using an in-house Perl script to obtain entries for proteins containing only a single predicted transmembrane domain (TMD). The signal peptide and TMD predictions applied to generate the LOCATE database have been estimated to correctly class >92% of proteins as ‘soluble cytosolic’, ‘soluble secreted’, ‘type I’, ‘type II’, ‘multispan’(42). These entries for single-pass transmembrane proteins were then parsed to find data pertaining to the position of the TMD, its topology, any external identifiers, subcellular locations from gene ontology (GO) identifiers and known motifs. The data were entered into a relational MySQL database as separate data sets for human and mouse. The Ensembl Perl API was then used to obtain known mouse orthologs for each human protein and to generate a linking table for the two data sets. A web interface was created to allow searches for human proteins containing TMDs of a given topology containing or not containing either known motifs or user-defined motifs. Links from the human hits to mouse orthologues are available in the output.

## Supporting Information

**Figure S1: Immunofluorescence double labelling for CD8 constructs and either early endosomal (EEA1) or late endosomal/lysosomal (CD63) markers.** Immunofluorescence on stably transfected, PFA-fixed, 0.1% TX-100 permeabilized cells was carried out using a rat mAb against CD8 (Abcam) and either mouse anti-EEA1 (Transduction Laboratories; A) or mouse anti-CD63 antibody (Biodesign International; B). Structures containing both CD8 and EEA1 or CD63 are marked with solid arrows; structures that contain CD8, but are devoid of EEA1 or CD63, are marked with barbed arrows.

**Figure S2: Quenching of Alexa Fluor 488-labelled anti-CD8 by anti-Alexa Fluor 488.** HeLa cells were transfected with wtCD for 24 h. The cells were harvested, incubated in the presence of anti-CD8 Alexa Fluor 488 antibody at 4°C, washed, and incubated in the presence (blue histograms) or absence (green histograms) of the anti-Alexa Fluor 488 quenching antibody. Quenching resulted in the loss of the 96% of the Alexa Fluor 488 fluorescence.

**Figure S3: CD8 chimeras isolated from the library with less efficient internalization signals.** A) Other cytoplasmic tail inserts (in addition to those shown in Figure 3) that promoted internalization of anti-CD8. Vector sequences are shown in italics. The nomenclature refers to the first three residues of the insert. B) The antibody uptake assay (see Figure 1) on cells transiently transfected with the CD8 chimeras.

**Figure S4: Western blotting to test the efficiency of clathrin and µ2 depletion by siRNA.** Blots of homogenates of control and siRNA-treated cells were probed with an affinity-purified rabbit polyclonal antiserum directed against clathrin heavy chain (43), a mouse monoclonal antibody against µ2 (anti-AP50; Transduction Laboratories) and a goat anti-EF-2 polyclonal antibody (Santa Cruz Biotechnology), followed by goat anti-rabbit, rabbit anti-mouse or rabbit anti-goat secondary antibodies conjugated to horseradish peroxidase (Sigma-Aldrich), and detected by luminescence using ECL Plus reagents (GE Healthcare).

**Figure S5: Vector used to generate CD8 library.** A) DNA sequence encoding cytosolic portion of the vector and protein sequence in three reading frames. The size-selected *Sau3A*I-digested yeast genomic DNA was ligated into the vector cut with *BamH*I enzyme. B) Internalization efficiency of the vector. HeLa cells were transfected with the pIRESneo2 vector expressing wtCD8 or a CD8 chimera with a modified tail containing a *BamH*I site for generation of the CD8 library. The antibody uptake assay was performed as described in Figure 2b,c.

**Table S1:** List of proteins with YXXXΦN sequence in the cytosolic tail.
