# Supplementary material for: A Screen for Endocytic Motifs
Source: Traffic. 2010 Apr 5;11(6):843–55. doi: 10.1111/j.1600-0854.2010.01056.x (PMC2882754; doi:10.1111/j.1600-0854.2010.01056.x)
Supplement: Supplementary file 7 [file tra0011-0843-SD6.pdf]

Table S1. *List of proteins with YXXXΦN sequence in the cytosolic tail.*

PrimaryID	Function
ENSP00000334828	CUB and sushi multiple domains 1 protein
NP_000132	K-sam-IIH3
AAH14484	TOR1A protein
NP_075261	fibroblast growth factor receptor 2 isoform 5 precursor
ENSP00000373212	ADAMTS-9 precursor (EC 3.4.24.-) (A disintegrin and metalloproteinase with thrombospondin motifs 9) (ADAM-TS 9) (ADAM-TS9).
ENSP00000215069	Uncharacterized protein C19orf15 precursor.
ENSP00000368317	Neuronal cell adhesion molecule precursor (Nr-CAM) (NgCAM-related cell adhesion molecule) (Ng-CAM-related) (hBravo).
NP_000104	torsin A
ENSP00000286301	
NP_075417	fibroblast growth factor receptor 2 isoform 10 precursor
ENSP00000369550	Retinal guanylyl cyclase 1 precursor (EC 4.6.1.2) (Guanylate cyclase 2D, retinal) (RETGC-1) (Rod outer segment membrane guanylate cyclase) (ROS-GC).
ENSP00000266744	achaete scute protein
NP_000777	cytochrome P450, family 51
ENSP00000354228	calcium channel, voltage-dependent, alpha 2/delta subunit 2 isoform b
NP_005205	CTLA4
NP_075264	fibroblast growth factor receptor 2 isoform 8 precursor
NP_075263	fibroblast growth factor receptor 2 isoform 7 precursor
XP_938838	RW1 protein isoform 5
ENSP00000310565	C18orf4 protein
ENSP00000366603	hTGN51
NP_075419	fibroblast growth factor receptor 2 isoform 12 precursor
NP_054699	receptor tyrosine kinase
NP_000519	mannosidase, alpha, class 2B, member 1 precursor
ENSP00000265342	hypothetical protein
ENSP00000353851	Torsin A precursor (Torsin family 1 member A) (Dystonia 1 protein).
ENSP00000295754	TGF-betaIIR alpha
ENSP00000261304	galactosylceramidase isoform a precursor
ENSP00000362125	Protocadherin-19 precursor.
ENSP00000373211	ADAMTS-9 precursor (EC 3.4.24.-) (A disintegrin and metalloproteinase with thrombospondin motifs 9) (ADAM-TS 9) (ADAM-TS9).
ENSP00000321821	CYP4F12 protein
ENSP00000365820	Low-density lipoprotein receptor-related protein 5 precursor.
NP_891550	ADAM metallopeptidase with thrombospondin type 1 motif, 9 preproprotein
NP_075262	fibroblast growth factor receptor 2 isoform 6 precursor
ENSP00000373213	ADAMTS-9 precursor (EC 3.4.24.-) (A disintegrin and metalloproteinase with thrombospondin motifs 9) (ADAM-TS 9) (ADAM-TS9).
ENSP00000358765	Trophoblast glycoprotein precursor (5T4 oncofetal trophoblast glycoprotein) (5T4 oncotrophoblast glycoprotein) (5T4 oncofetal antigen) (M6P1).
BAC04415	
ENSP00000312001	leucine rich repeat neuronal 3
ENSP00000252483	Poliovirus receptor-related protein 2 precursor (Herpes virus entry mediator B) (HveB) (Nectin-2) (CD112 antigen).
AAH28933	FCRL3 protein
NP_006197	platelet-derived growth factor receptor alpha precursor
ENSP00000350356	NT-3 growth factor receptor precursor (EC 2.7.10.1) (Neurotrophic tyrosine kinase receptor type 3) (TrkC tyrosine kinase) (GP145-TrkC) (Trk-C).
ENSP00000358061	Fibroblast growth factor receptor 2 precursor (EC 2.7.10.1) (FGFR-2) (Keratinocyte growth factor receptor 2) (CD332 antigen).
ENSP00000294304	LDL receptor member LR3

NP_001014796	discoidin domain receptor family, member 2 precursor
ENSP00000364785	Ephrin type-A receptor 2 precursor (EC 2.7.10.1) (Tyrosine-protein kinase receptor ECK) (Epithelial cell kinase).
ENSP00000311265	Complement C1q tumor necrosis factor-related protein 1 precursor (G protein-coupled receptor-interacting protein) (GIP).
ENSP00000317128	plexin D1
NP_075418	fibroblast growth factor receptor 2 isoform 11 precursor
NP_004516	gp330 precursor
ENSP00000365759	Epithelial discoidin domain-containing receptor 1 precursor (EC 2.7.10.1) (Epithelial discoidin domain receptor 1) (Tyrosine kinase DDR) (Discoidin receptor tyrosine kinase) (Tyrosine-protein kinase CAK) (Cell adhesion kinase) (TRK E) (Protein-tyrosine ki
ENSP00000261349	LDL receptor-related protein 6
ENSP00000364643	Long palate, lung and nasal epithelium carcinoma-associated protein 3 precursor (Ligand-binding protein RYA3).
ENSP00000303713	mucin 15
NP_054700	discoidin domain receptor family, member 1 isoform c
NP_004434	ephrin receptor EphB3 precursor
ENSP00000367433	Adherens junction-associated protein 1 (Membrane protein shrew-1).
ENSP00000321345	Interleukin-23 receptor precursor (IL-23R).
ENSP00000216286	osteonidogen
XP_938643	hypothetical protein LOC57186 isoform 3
NP_075258	fibroblast growth factor receptor 2 isoform 2 precursor
